# Knowledge, attitudes and determinants of exclusive breastfeeding practice among Ghanaian rural lactating mothers

**DOI:** 10.1186/s13006-016-0071-z

**Published:** 2016-05-17

**Authors:** Victor Mogre, Michael Dery, Patience K. Gaa

**Affiliations:** Department of Health Professions Education and Innovative Learning, School of Medicine and Health Sciences, University for Development Studies, Box TL 1883, Tamale, Ghana; Department of Community Nutrition, School of Allied Health Sciences, University for Development Studies, Box TL 1883, Tamale, Ghana; Nutrition Unit, Lawra District Hospital, Box 19, Upper West Region, Lawra Ghana

**Keywords:** Exclusive breastfeeding, Infants, Children under-five, Lactating mothers, Rural, Ghana

## Abstract

**Background:**

The practice of exclusive breastfeeding (EBF) is influenced by maternal knowledge and attitudes as well as socio-demographic and cultural factors. This study assessed knowledge, attitudes and practice of EBF among rural lactating mothers with infants aged 0–6 months. Factors associated to the practice of EBF were also investigated.

**Methods:**

This cross-sectional study was conducted among 190 rural lactating mothers with infants aged 0–6 months seeking postnatal care at a health centre in Ghana. All data was collected using a questionnaire that contained both closed and open ended questions.

**Results:**

About 26 % (*n* = 50) of the mothers were unable to correctly define EBF. The majority (92.6 %, *n* = 176) of the mothers said they felt good to EBF for 6 months, to breastfed on demand (99.5 %, *n* = 189) and did not have difficulties EBF (90 %, *n* = 171). Despite the generally positive attitude towards EBF, 42 % (*n* = 79) of the mothers did not EBF their babies. These mothers did not practice EBF because they misunderstood certain signs of the child to mean wanting to eat food or drink water, regarded breastmilk to be inadequate to meet the nutritional needs of the child and misunderstood healthcare professionals’ EBF advice. Higher maternal education was associated with higher likelihood of EBF (OR 3.5; 95 % CI 1.6, 7.7; *p* = 0.002). Mothers whose babies were younger than 3 months were more likely to EBF (OR 12.0; 95 % CI 4.4, 32.5; *p* < 0.001) than those having babies aged ≥ 3 months. Furthermore, higher knowledge of EBF was associated with the likelihood of EBF (OR 5.9; 95 % CI 2.6, 13.3; *p* < 0.001).

**Conclusion:**

Mothers’ knowledge and attitudes towards EBF were favourable but practice of EBF was suboptimal. This study adds additional evidence that knowledge of EBF, child’s age and maternal level of education are important determinants of the practice of EBF. Beyond dissemination of health messages, healthcare professionals should pay more counselling attention to less educated mothers, and also older children’s caregivers.

## Background

One of the most effective strategies for reducing infant morbidity and mortality in resource limited settings (i.e. human and infrastructural constraints) is the promotion of exclusive breastfeeding (EBF) for the first 6 months of the infant’s life [1–4]. Studies have established the important role of EBF for growth, immunity and prevention of illness in infants [5, 6]. According to the Lancet’s Series on Child Survival, increasing breastfeeding prevalence to optimal levels could reduce 13 % of all child deaths in low income countries [7]. Suboptimal breastfeeding was ranked by the Global Burden of Diseases, Injuries and Risk Factor Study to be the second largest risk factor for children under five, accounting for the loss of 47.5 million Disability Adjusted Life Years (DALYs) in 2010 [8]. Sub-Saharan Africa has been the worst affected with the highest proportion of disease burden associated to suboptimal breastfeeding [8]. The World Health Organization (WHO) recommends timely initiation of breastfeeding within the first hour of birth, exclusively breastfeeding up to the age of 6 months and continued breastfeeding through to 24 months together with appropriate complementary feeding [7, 9].

To promote EBF, Ghana adopted the Baby-Friendly Hospital Initiative (BFHI) in 1991 and subsequently formed a BFHI authority to oversee its implementation, development of breastfeeding policy and training of health workers to promote and support the practice of breastfeeding [10, 11]. Furthermore, Ghana enacted and implemented the Ghana Breastfeeding Promotion Regulation 2000 otherwise known as Legislative Instrument [LI] 1667) to curb the aggressive marketing of breastmilk substitutes [12, 13].

Similar to several countries in the sub-Saharan region, these have not yielded the desired impact as the practice of exclusive breastfeeding is still low [14, 15]. In 2008, the Ghana Demographic and Health Survey (GDHS) estimated Ghana’s EBF rate to be 63 % [16]. Forty-six percent of Ghanaian children aged less than 6 months were exclusively breastfed in 2011 [17]. Obviously these estimates fall short of the WHO’s recommendation of 90 % coverage [7].

Mother's knowledge and attitudes towards exclusive breastfeeding as well as family pressures, maternal level of education, knowledge, attitude, socio-cultural tradition, maternal age, marital status, family income/social class, place of delivery, and time of initiation of first breastfeeding have been previously cited for the current situation [10, 15, 18–28]. Most of these studies were done predominantly among mothers in the urban settings of Ghana. Evidence on the practice and determinants of exclusive breastfeeding among rural lactating mothers is limited. It is thus imperative to explore these mothers’ knowledge of and attitudes towards EBF and determinants of the practice of EBF. Evidence from this study may further increase understanding of the practice and associated factors of EBF among rural mothers. Understanding the knowledge, attitude and practice of EBF and determinants of practice may be a necessary step to help improve infant feeding practices among rural lactating mothers as a means of reducing infant morbidity and mortality. Furthermore, findings from this study may be used as a basis for the design of future EBF promotion programs to improve the knowledge, attitudes and practice of exclusive breastfeeding among rural mothers.

This study identified gaps in knowledge, attitudes and practice of EBF among rural lactating mothers with infants aged 0–6 months. It further explored the determinants of the practice of EBF in this sample.

## Methods

### Study area and participants

This cross-sectional study was carried out at the Tuna health centre, a sub-district health facility from January to July 2015. Tuna is a rural co-district capital of the Sawla-Tuna-Kabla district in the northern region of Ghana. Over 80 % of the communities that seek for health care from the Tuna Health Centre are rural. Services offered by the health centre include maternal and child health services such as antenatal, postnatal, growth monitoring, family planning, vaccinations, immunizations and health promotion as well as general preventive and curative services.

This study considered all mothers and/or care givers that attended the antenatal clinic of the Tuna Health Centre with apparently healthy infants aged 0–6 months during the study period. The mother-infant pairs that met the inclusion criteria were enrolled into the study.

### Recruitment and data collection procedures

Recruitment and data collection procedures were done by the second author. The health centre was visited during the study period on days that were scheduled for postnatal care. The mother-infant pairs were approached while they waited to receive their postnatal care, to introduce the study to them and seek their consent to participate. Those who agreed to participate were taken through the consent processes, explaining to them the benefits and risks of participating in the study. Voluntary participation was encouraged. In a secluded area at the health centre, a questionnaire was administered face-to-face in the local dialect to the mothers who agreed and consented to participate in the study. All data collection procedures were carried out within 15–20 min.

In all, 217 mother/care giver-infant pairs were approached in which 200 agreed and consented to participate in the study. Ethical approval (Reference no. = CMN/1140/11) of the study was granted by the Ethics Review Committee of the School of Allied Health Sciences of the University for Development Studies, Tamale, Ghana.

### Inclusion criteria

Mothers with infants aged 0–6 months of age who presented to the health centre without any acute or chronic illness were eligible to participate in the study.

### Exclusion criteria

Infants aged older than 6 months, mothers and infants aged 0–6 months who were HIV exposed, infected or whose HIV status was not known, and infants aged 0–6 months who presented to the health centre with acute and chronic illness were not eligible to participate.

### Knowledge, attitude and practice (KAP) questionnaire

A questionnaire consisting of both closed and open ended questions was used to collect all data on socio-demographic factors (maternal age, infant’s age, parity, as well as maternal educational level, and marital, occupational and religious statuses), knowledge on exclusive breastfeeding (EBF), attitude towards EBF, and practice of EBF. The open ended questions were included to gain understanding of why mothers gave a specific answer. Items for the knowledge, attitude and practice of EBF scales of the questionnaire were adapted from the Food and Agriculture Organization of the United Nations (FAO) guidelines for assessing nutrition-related knowledge, attitudes and practices (KAP) manual. This manual contains guidelines that serve as a reference guide and practical tools for undertaking high quality evaluation of nutrition and health related knowledge, attitudes and practices at the community level [29]. This manual has 13 module questionnaires capturing data on important knowledge, attitudes and practices related to 13 most common nutrition issues such as feeding infants (0–6 months), feeding young children (6–23 months), diet of school-aged children and among others. Based on the aims and objectives of this study, the questionnaire pertaining to feeding infants younger than 6 months was adapted for this study. The FAO questionnaire has been field tested in several countries to ensure validity, readability, ease of administration and is less burdensome on respondents [29].

The knowledge scale of the questionnaire consisted of 13 questions assessing mothers’ understanding and intellectual capacity to recall the benefits of EBF, duration of EBF, and how to improve breastmilk supply. Each correct response was accorded a point and no point for each wrong response. A knowledge score was generated for each mother based on the number of correctly answered questions.

The attitude scale of the questionnaire consisted of six items assessing mothers’ perceived benefits and barriers to exclusively breastfeed and breastfeeding on demand as well as self-confidence in expressing and storing breastmilk. Mothers were asked to rate their response on a three-point Likert scale measuring the intensity of the mothers attitudes [29]. Each attitude item had three response options including a positive response, a middle option capturing uncertain attitudes and a negative option.

The practice scale consisted of six items that assessed mothers’ practice of EBF relating to the following: recall of EBF in the last 24 h, mode of breastfeeding, who gave and what kind of food was given to the baby in the mothers absence, introduction of liquids (i.e. plain water, infant formula, tinned milk, powdered or fresh animal milk, juice/juice drinks, clear broth, yogurt, porridge, herbal teas, solid/marshy foods). The mothers’ answers to these questions were used to determine the practice of EBF. The form and nature of these items were provided by the United Nations Children’s Fund (UNICEF) Multiple Indicator Cluster Surveys and the Demographic and Health Surveys [29].

In accordance with the FAO guidelines, practice of EBF preceded the knowledge and attitude test. For purposes of content validity and appropriateness for the local context, items of the questionnaire were reviewed by a team of nutrition experts. This resulted in the addition of local foods commonly given to infants in the study setting. The questionnaire was pretested on a sample of 10 mothers with infants aged 0–6 months for purposes of comprehension, readability and easiness of administration.

### Statistical analysis

All data were entered into Microsoft Excel and analysed using SPSS version 20. Both qualitative and quantitative data was collected and analysed accordingly. Responses for all the open-ended questions (qualitative data) were read and re-read by all the authors. The responses were coded by the second author and the results reviewed by VM and PKG. Common themes were identified through discussions and reflections. All quantitative data were analysed using descriptive statistics of mean for continuous variables, and frequencies and percentages for categorical variables. Cross tabulation and chi-square tests were used to determine univariate associations.

To determine factors associated with the practice of EBF, a multivariate logistic regression was executed. The dependent variable of the logistic model was the practice of EBF. Only variables that were significantly associated to the practice of exclusive breastfeeding in the univariate analysis were included into the logistic regression model. Results are presented as odds ratios and their respective confidence intervals at 95 %. In all analysis a *p*-value of <0.05 was considered statistically significant.

## Results

Presented in Table 1 are the general characteristics of the mothers. With a mean age of 27.27 ± 5.87 years, the majority of the mothers were employed 79.5 %, *n* = 151), currently married (93.2 %, *n* = 177) and have had some form of formal education (61 %, *n* = 1160). The ages of the infants ranged between 1 and 6 months (mean ± SD = 3.29 ± 1.60 months).

**Table 1:** General characteristics of the study participants (*n* = 190)

Variable	Frequency (%)
Mother’s age category (in years)	
≤20	15 (7.9)
21–30	134 (70.5)
31–40	37 (19.5)
>40	4 (2.1)
Child’s age category (in months)	
1–3	104 (54.7)
4–6	86 (45.3)
Mean ± SD parity	2.23 ± 1.44
Employment status	
Employed	151 (79.5)
Unemployed	39 (20.5)
Marital status	
Married	177 (93.2)
Unmarried/single	13 (6.8)
Religious status	
Christianity	104 (54.7)
Islamic	84 (44.2)
ATR	2 (1.1)
Educational level	
No education	74 (38.9)
Primary education	73 (38.4)
Secondary education	43 (22.6)

*ATR* African Traditional Religion; *SD* standard deviation

The mothers’ knowledge in aspects of EBF is presented in Table 2. About 26 % (*n* = 50) of them were unable to define EBF; 66 % (*n* = 33) defined EBF as giving the child breastmilk and water and the others did not have an idea. Twenty two percent (*n* = 41) of the mothers said breastmilk only is not sufficient to meet the nutritional needs of the child. The reasons they offered for holding this view were that the child may not be satisfied and could die if fed with only breastmilk for 6 months. Others also had the opinion that the child also feels thirsty and should be given water to drink. The majority (*n* = 169, 88.9 %) of the mothers did not know that breastmilk could be expressed, stored safely and given to the child in times of the mother’s absence.

**Table 2:** Mothers’ knowledge of exclusive breastfeeding (*n* = 190)

Variable	Frequency (%)
First food for the newborn is breastmilk	186 (97.9)
Exclusive breastfeeding is giving the child breastmilk for the first 6 months	140 (73.7)
Babies should take only breastmilk for the first 6 months of their life	149 (78.4)
Breastmilk only is sufficient for the baby’s first 6 months of life	149 (78.4)
The baby should be breastfed on demand	177 (93.2)
Has knowledge on the benefits of exclusive breastfeeding to the baby	182 (95.8)
Exclusive breastfeeding is beneficial to the mother	168 (88.4)
Breastmilk supply can be sustained by having good nutrition/eating well	162 (85.3)
In times of absence the baby can continue to be exclusively breastfed by expressing breastmilk and storing	21 (11.1)
Health personnel can assists in overcoming breastfeeding difficulties	131 (68.9 %)
Knowledge category^a^	
High (>70 %)	87 (45.8)
Mean ± SD Knowledge score (maximum score = 20)	13.95 ± 2.83

^a^According to the FAO guidelines thresholds suggestive of a nutrition intervention, a knowledge score of ≤ 70 % is considered urgent for nutrition intervention. All mothers who scored > 70 % in the knowledge test was considered to have a high level of knowledge and those scoring ≤ 70 % were considered as having a low level of knowledge

Regarding how to overcome breastfeeding difficulties, 8.9 % (*n* = 17) of the mothers said herbs/drugs could be taken to overcome the difficulty; 6.8 % (*n* = 13) said breastfeeding should be stopped; 11.1 % (*n* = 21) said breastfeeding should be continued and 4.2 % (*n* = 8) did not know what to do.

Presented in Table 3 is the mothers’ attitude towards the practice of EBF. Lack of time and difficulty sleeping were the reasons given by 6.5 % (*n* = 13) of mothers who said they did not feel good about exclusively breastfeeding for 6 months and 9.5 % (*n* = 19) said they had difficulty breastfeeding on demand. The majority (81.1 %, *n* = 154) of the mothers said they were not confident to express and store breastmilk. The reasons given by the mothers were that the expressed breast milk could get contaminated, may lose its nutrients and it is a taboo to express human breastmilk. Also, some mothers expressed their inability to express enough breastmilk to satisfy the child, while others said they have never witnessed anyone expressing breastmilk for them to learn from.

**Table 3:** Mothers’ attitudes towards exclusive breastfeeding (*n* = 190)

Variable	Frequency (%)
Feels good to exclusively breastfeed my baby for 6 months	176 (92.6)
I find it difficult exclusively breastfeeding my baby for 6 months	22 (11.6)
I feel good breastfeeding my baby on demand	189 (99.5)
I find it difficult breastfeeding my baby on demand	19 (10.0)
I feel confident expressing my breastmilk to be given to my baby if am not available	36 (18.9)
Mean ± SD Attitude Score (Maximum score = 15)	11.74 ± 2.20
^a^Attitude category	
Positive	157 (82.6)
Less positive	33 (17.4)

^a^According to the FAO guidelines thresholds suggestive of a nutrition intervention, an attitude score of ≤ 70 % is considered urgent for nutrition intervention. All mothers who scored > 70 % in the attitude test were considered to have positive attitude and those scoring ≤ 70 % were considered to be less positive

Almost 42 % (*n* = 79) of the mothers did not exclusively breastfeed their babies. These mothers reported giving the infants water, porridge and other liquids. These mothers said they did not practice EBF because their babies usually showed signs of wanting to eat food or drink water. Others also misunderstood the advice of healthcare professionals’ to mean water should be avoided but foods could be given. Furthermore, some mothers said foods such as fresh cow milk are similar to breastmilk and could be given to the child. The majority (94.5 %, *n* = 189) of mothers reported never leaving their babies at home. Among those who reported ever leaving their babies at home, only one mother said the baby was fed with expressed breastmilk during her absence and the rest said the baby was fed with infant formula and/or other foods.

The characteristics of mothers who do and do not practice EBF are presented in Table 4. Mothers who practiced EBF were more likely to have high knowledge in EBF and positive attitudes towards EBF than their counterparts. Furthermore, they were more likely than their counterparts to report having infants younger than 3 months and high level of education.

**Table 4:** Characteristics of mothers who do and do not practice exclusive breastfeeding (*n* = 190)

	Exclusively breastfeeds		
Variable	Yes (*n* = 111)	No (*n* = 79)	*p*-value
Age of mother (in years)			
<30	45 (40.5 %)	27 (34.2 %)	0.230
≥30	66 (59.5 %)	52 (65.8 %)	
Child’s age (in months)			
<3	64 (57.7 %)	6 (7.6 %)	<0.001
≥3	47 (42.3 %)	73 (92.4 %)	
Mother’s employment status			
Employed	85 (76.6 %)	66 (83.5 %)	0.161
Unemployed	26 (23.4 %)	13 (16.5 %)	
Mothers’ educational level			
Low educational level	34 (30.6 %)	40 (50.6 %)	0.004
High educational level	77 (69.4 %)	39 (49.4 %)	
Parity			
1	42 (37.8 %)	28 (35.4 %)	0.428
>1	69 (62.2 %)	51 (64.6 %)	
Marital status			
Not married	8 (7.2 %)	5 (6.3 %)	0.528
Married	103 (92.8 %)	74 (93.7 %)	
Mothers’ religion			
Christianity	60 (54.1 %)	44 (55.7 %)	0.221
Islamic	51 (45.9 %)	33 (41.8 %)	
African traditional religion	0 (0.0 %)	2 (2.5 %)	
Mothers’ knowledge of exclusive breastfeeding			
High	72 (64.9 %)	15 (19.0 %)	<0.001
Low	39 (35.1 %)	64 (81.0 %)	
Mothers’ attitude towards exclusive breastfeeding			
Positive	99 (89.2 %)	58 (73.4 %)	0.004
Less positive	12 (10.8 %)	21 (26.6 %)	

To identify factors associated with the practice of exclusive breastfeeding, a multivariable logistic regression model was executed and the findings presented in Table 5. Having infants younger than 3 months, high maternal educational level and a thorough knowledge in EBF remained significantly associated to the practice of EBF.

**Table 5:** Multivariate determinants of exclusive breastfeeding (*n* = 190)

Variable	B	AOR (95 % CI)	*p*-value
Child younger than 3 months	2.49	12.02 (4.44, 32.54)	<0.001
High maternal level of education	1.24	3.47 (1.55, 7.75)	0.002
High level of maternal knowledge of EBF	1.77	5.87 (2.59, 13.26)	<0.001
Positive maternal attitude	0.63	1.88 (0.69, 5.11)	0.216

Nagelkerke R square = 0.495

## Discussion

In this study we assessed knowledge in, attitude towards EBF and its practice among rural lactating mothers with infants aged 0–6 months. Determinants of the practice of EBF in this sample were also evaluated. The mothers’ knowledge of EBF was generally high, although some notable gaps were identified. The attitude of the mothers towards EBF was also positive, however the practice of EBF was found to be lower than desired. Factors that were found to be associated to the practice of EBF included maternal level of education, child’s age and having high knowledge of EBF.

### Gaps in mothers’ knowledge, attitudes and practice of EBF

Misconceptions relating to duration of EBF and the inadequacy of breastmilk to meet their child’s nutritional needs were noted. Most mothers also had inadequate knowledge of the maternal benefits of exclusive breastfeeding. Similar misconceptions and inadequacies of knowledge have been reported previously [21, 28]. Emphasising on the maternal benefits of EBF could help encourage mothers to exclusively breastfeed their infants.

An important finding of this study was that most mothers were more likely to consult relatives and significant others to overcome breastfeeding challenges instead of consulting healthcare providers. Although consulting relatives and significant others may not be inappropriate, the accuracy and quality of advice and support given may not be guaranteed making mothers prone to inappropriate advice and support. Postnatal visits to the health centre are opportunities that healthcare professionals could rely upon to encourage mothers to seek support in times of difficulties. Building on the current knowledge and the use of active teaching and learning strategies such as discussions, lectures, slides, and presentations could be adopted to bridge these gaps in knowledge.

Although the mothers’ attitude towards EBF was generally positive, a large proportion of them were uncomfortable with the idea of expressing and storing breastmilk. Taking cognizance of the FAO/UN KAP threshold levels this requires urgent attention as the mothers scored lower than 70 % for their attitude towards the expressing of breastmilk. This presents an important challenge for mothers who will not always be at home due to one reason or the other, to continuously breastfeed exclusively. Interventions should be designed to increase mothers’ confidence and dispel their misconceptions regarding expressing and storing of breastmilk. This is especially necessary for employed mothers who may have to leave their babies at home to enable them work and exclusively breastfeed at the same time.

Fifty-eight percent of the mothers practiced EBF. This is higher than the 46 % of Ghanaian children aged less than 6 months being exclusively breastfed in 2011 [17] but lower than the 64 % reported by Tampah-Naah and Kumi-Kyeremee [10] using data from the 2008 Ghana Demographic and Health Survey (GDHS). The prevalence of EBF found in this study is far below the WHO recommended prevalence of 90 % [7] demonstrating a wide gap between the desired and the actual practice of exclusive breastfeeding. The low prevalence of exclusive breastfeeding could be attributed to misconceptions regarding the inadequacy of breastmilk to meet the nutritional needs of the child, misunderstanding certain signs of the child to mean she/he is showing signs of wanting food to eat and misunderstanding healthcare professional’s advice. Similar misconceptions have been reported previously in rural Ghana and in other West African countries [15, 21, 27, 28]. Education on exclusive breastfeeding is usually disseminated to mothers in the form of health talks by midwives, nurses or nutritionists during antenatal and postnatal clinic visits. As suggested by previous studies [1, 30, 31], the findings of this study calls for an evaluation of the content of such health talks and the mothers understanding of the messages provided to them as significant gaps in knowledge of exclusive breastfeeding.

### Determinants of the practice of exclusive breastfeeding

Mothers with higher level of education were more likely to report higher practice of exclusive breastfeeding than their counterparts. Maternal level of education has been found to be an important determinant of infant feeding practices in several studies in Ghana [18, 26]. Mothers with higher levels of education may be able to comprehend and appreciate the benefits of EBF to their infants and more motivated to practice it [1]. Suggestively, exclusive breastfeeding promotion programs should be made more appealing to mothers who have lower levels of education. For instance, healthcare providers could emphasis on the fact that exclusive breastfeeding is not only beneficial to the infant but also for the mother regarding delayed return of ovulation, reduction in the risk of developing breast cancer and protection against postpartum bleeding [18].

Another important determinant of the practice of exclusive breastfeeding was the age of the child. Significantly, mothers with babies younger than 3 months were more likely to practice exclusive breastfeeding compared to those having babies aged 3 months or older. Similar findings have been reported previously in Ghana and other parts of West Africa [26, 32, 33]. As the age of the child increases, mothers are more likely to begin to introduce other foods as they perceive that breastmilk alone might not be sufficient to meet the nutritional needs of the child. These finding suggests that healthcare professionals should pay special attention to lactating mothers as the baby grows, by encouraging and supporting them to overcome barriers that may prevent them from exclusively breastfeeding. Given the fact that most mothers may return to work as the child grows older, and their lack of confidence to express and store breastmilk, it is plausible that mothers may begin to introduce other foods to the child in order to have time to work and attend to other activities [31]. Misconceptions of mothers regarding expressing and storing breastmilk should be identified and given attention in future EBF promotion programs. Mothers’ should be encouraged and supported to gain the appropriate knowledge and confidence to be able to express and store breastmilk that could be used to feed the child while they were at work. Notwithstanding the above, we recommend that future studies should explore the contributing factors responsible for the decrease in the practice of exclusive breastfeeding as the baby grows older in this setting.

Another factor that was found to be associated to the practice of exclusive breastfeeding was having knowledge of EBF. Mothers who had higher knowledge were more likely than their counterparts with low knowledge in EBF to report practicing it. Similar to our findings, studies that report high maternal knowledge on EBF also report high prevalence of the practice of exclusive breastfeeding [34–36] and the reverse is true [37, 38]. Lack of knowledge of the benefits of breastfeeding has been reported to contribute to the low level of exclusive breastfeeding practice in Sub-Saharan Africa [31].

### Strengths and limitations

The items of the questionnaire of this study have been previously validated improving the credibility of our findings. Furthermore, the questionnaire included both open and closed ended items providing a better understanding of the factors associated to the practice of exclusive breastfeeding of the mothers. It also enabled us to offset the weakness of following only quantitative and qualitative approaches.

This study is not without limitations. Its cross-sectional nature makes it difficult to establish causality. This study was health centre based involving only mothers that sought for postnatal care at the health centres. As such the findings of this study may not be representative of the situation of exclusive breastfeeding in the entire community. It is therefore imperative to conduct a community-based study to explore the knowledge, attitude and practice of exclusive breastfeeding at the community level. Furthermore, the use of non-probability sampling also affects the generalizability of our findings. Our data collection method is also prone to recall and social desirability bias.

## Conclusion

The lactating mothers’ knowledge of and attitudes towards EBF were generally favourable. However, their practice of EBF was suboptimal. Mothers’ misconceptions and misunderstanding of EBF messages may play an important role in determining the practice of EBF. Maternal knowledge, maternal level of education and age of the child may also be important in promoting the practice of EBF. Healthcare professionals should go beyond the mere dissemination of information to encouraging and helping mothers to overcome barriers of practicing EBF.
